# Serum Calcium and Magnesium Levels in Women with Uterine Fibroids at a University Teaching Hospital in Southwest Nigeria: A Comparative Cross-Sectional Study

**DOI:** 10.21203/rs.3.rs-2877359/v1

**Published:** 2023-05-04

**Authors:** Fatimah ADEBOJE-JIMOH, Kehinde Sharafadeen OKUNADE, Gbenga OLORUNFEMI, Joseph Ayodeji OLAMIJULO

**Affiliations:** Lagos University Teaching Hospital; Lagos University Teaching Hospital; University of Witwatersrand; Lagos University Teaching Hospital

**Keywords:** Ca2+, Leiomyoma, Nigeria, Trace elements, Ultrasound scan

## Abstract

**Background:**

Studies have suggested the potential roles of serum trace elements such as calcium and magnesium in the development of uterine fibroids.

**Aims:**

This study compared magnesium and calcium serum levels in reproductive-age women with and without uterine fibroids in Lagos, Southwest Nigeria.

**Methods:**

A comparative cross-sectional study of 194 parity-matched women with or without a sonographic diagnosis of uterine fibroids enrolled at a university teaching hospital in Lagos, Southwest Nigeria. Participants’ sociodemographic, ultrasound, and anthropometric information as well as the estimated serum levels of calcium and magnesium were collected for statistical analyses.

**Results:**

This study found significant negative associations between low serum calcium levels and uterine fibroids (adjusted odds ratio= 0.06; 95% CI: 0.004, 0.958; p=0.047), uterine size (p=0.004), and the number of fibroid nodules (p=0.030). However, no significant association was observed between serum magnesium levels and uterine fibroids (p=0.341).

**Conclusion:**

The findings of this study suggest the promising role of calcium-rich diets and supplements in the prevention of uterine fibroids among Nigerian women. However, future longitudinal studies are required to further evaluate the potential role of these trace mineral elements in the development of uterine fibroids.

## Introduction

Uterine fibroid or leiomyoma is a benign smooth muscle tumour of the uterus that affects women of reproductive age [1]. It is the most common of all pelvic tumours in women [2] and has been postulated to occur in over 70% of women by the onset of menopause [1, 3]. Uterine fibroids are estimated to be clinically apparent in 25% of women of reproductive age and are severe enough to cause symptoms that require treatment in approximately 25% of these women [4, 5]. It accounts for 6.4% of all gynaecological ward admissions and 21.3% of all major gynaecological surgeries [6]. Some of the common features of symptomatic uterine fibroids include abnormal uterine bleeding, abdominal swelling, and pelvic pressure symptoms such as urinary frequency, incontinence, constipation, and tenesmus and pelvic pain [2, 7, 8]. It is regarded as a disease of public health importance due to its short-term disability, absenteeism and loss of work hours, impact on the quality of life, and psychological effects on affected women [7]. The annual societal costs of uterine fibroids in the United States range from $5.9 billion to $34.4 billion and these include the complications attributable to the tumours amount of work time lost and the costs of treatment [5].

Uterine fibroids are monoclonal tumours of uterine smooth muscle origin that consist of a large amount of extracellular matrix including collagen, fibronectin, and proteoglycan [9, 10]. Even though their pathogenesis is not known, there is considerable evidence to suggest the role of micronutrient deficiencies including minerals and trace elements in the growth of these benign tumours [7, 11, 12]. Available epidemiological data suggest that certain dietary components can influence the formation and growth of hormone-related diseases such as uterine fibroids [13]. A study conducted by Morakinyo and colleagues in 2015 [14] demonstrated the inadequacies of some mineral elements including calcium and magnesium relative to the recommended daily allowance in commonly consumed local diets in Nigeria. Calcium and magnesium are important co-factors in the synthesis and repair of DNA and the regulation of hormones that control tumour growth [15]. African women are often affected by a lack of optimal nutritional intake resulting in deficiencies of important minerals and trace elements thus suggesting their increased vulnerability to the formation and growth of uterine fibroids. However, there is currently a paucity of studies to determine the associations between serum calcium and magnesium levels among women of reproductive age group and uterine fibroid in our environment. This study was, therefore, conducted to compare the serum levels of calcium and magnesium in reproductive-age women with and without uterine fibroids and to determine the prevalence of these trace element deficiencies among women at the gynaecological outpatient clinics of a University Teaching Hospital in Southwest Nigeria.

## Patients and Methods

### Study design and setting

This was a comparative cross-sectional study carried out among reproductive-age women with and without uterine fibroids at a University Teaching Hospital in Southwest Nigeria between April and September 2021. The hospital is the teaching hospital of a College of Medicine, and it is an over 700-bedded facility located in an urban cosmopolitan setting that borders two major urban communities. It is the second largest tertiary health institution in Southwest Nigeria and acts mainly as a referral centre for other government-owned and private hospitals in the region. The gynaecology clinic of the hospital is an all-female clinic that runs from 2 pm to 6 pm on every weekday except Friday. The clinic days are run by 4 different units led by a minimum of 4 to 5 consultants per unit. An average of 18 new patients are seen at each gynaecology clinic out of which approximately 7 are diagnosed with uterine fibroids.

### Study population and eligibility criteria

The study population comprised of consenting reproductive age (15–49 years old) women with uterine fibroids and their parity-matched (± 2) comparison group without uterine fibroids at the study site. Women with uterine fibroids are defined as those with sonographic evidence of one or more uterine fibroid seedlings of at least 10mm in size. Excluded from the study were women on known magnesium and/or calcium metabolism-altering drugs such as antacids (such as proton pump inhibitors and H_2_ receptor blockers), aluminium preparations, diuretics, digitalis, anti-neoplastic agents and aminoglycosides; those with co-existing chronic medical conditions such as malignancies, hypertension, diabetes and thyroid dysfunction; and those on hormonal treatment (including oral contraceptives) and magnesium or calcium supplementation in the last three months before their enrolment. Also excluded were women who have had a previous myomectomy or hysterectomy.

### Study outcomes and sample size determination

The measures of the primary outcomes for the study were the mean differences in the serum levels of magnesium and calcium in women with and without uterine fibroids while the secondary outcomes were the prevalence of serum magnesium and calcium deficiencies in women with and without uterine fibroids and the correlations between serum magnesium and calcium levels and uterine sizes (cm) and numbers of uterine fibroid(s). The sample size [16] for each of the study groups (n = 97) was calculated using an effect size of 8.0mg/L for calcium levels [12] (being the analyte with the larger sample size) and a standard deviation of 1.9mg/L to achieve a power of 80% (0.842) at a type 1 error rate of 5% (1.96) with 95% confidence level while adjusting for a non-response or data-recording error rate of 10%.

### Participants’ enrolment and data collection

All the potentially eligible reproductive-age women attending the gynaecological out-patients clinics during the study period were screened for eligibility following which the purpose and procedures of the study were and their informed consent obtained. Consecutively consenting women were then enrolled by consecutive sampling after a screening pelvic ultrasonography at the Radiodiagnosis department of the study site until the sample size required for the two study groups was achieved. The ultrasound scan was performed using a Toshiba ultrasound unit XG SSA-58GA platinum series with the use of a transabdominal probe at a frequency of 3.0–5.0 MHz and the findings were interpreted in real-time by an experienced sonologist of at least the rank of a Year 5 postgraduate resident doctor in the Radiodiagnosis residency training programme. Information about prior ultrasound scan reports, if any, was not disclosed to the sonologist. A study proforma was then used to collect sociodemographic information (age (in years), occupation, parity, marital status, and educational status) and ultrasound findings (presence or absence of fibroids, uterine dimensions (in cm), fibroid sizes (in mm), and the number of fibroids). Following this, the participant’s anthropometric parameters such as weight (in kg) and height (in meters) were measured using a portable weighing scale mounted on a stadiometer with a movable headpiece to calculate the body mass index (BMI) (in kg/meter^2^) and then about four millilitres (4 mL) of venous blood were collected from each participant’s antecubital vein into a plain vacutainer bottle labelled with the participant’s unique identification code and then transported in an icepack at a temperature of minus 2 to 8 °C to the central research laboratory where the sample was centrifuged at 3000 rpm for 10 minutes and the supernatant decanted to separate the serum from the red cells. Following this, about 1 mL of serum was extracted and stored in cryogenic vials in a minus 20°C freezer until the final laboratory analysis.

### Laboratory analysis

Sample analysis of calcium and magnesium were performed using a colorimetric method with the Cobas c311 auto-analyzer machines manufactured by Roche in the United State of America [17]. The relevant trace element reagent kits contained controls to minimize bias within the intra- and inter-batch analysis.

#### Magnesium estimation

The principle is based on the reaction of magnesium with xylidyl blue in the reagent in an alkaline solution containing EGTA (ethylene glycol tetraacetic acid). The EGTA masks the calcium in the sample. In an alkaline solution, magnesium forms a purple complex with xylidyl blue and diazonium salt. The magnesium concentration is measured photometrically via the decrease in the xylidyl blue absorbance. The absorbance was measured at 505–600nm wavelength with a detection limit for serum magnesium level is approximately 0.1mmol/l. The expected value for serum magnesium levels for a normal female adult is 0.66–1.07mmol/L.

#### Serum calcium estimation

The principle is based on calcium ions reaction with 5-nitro-5’-methyl-(1,2-bis(o-amino phenoxy) ethane-N, N, N, N–tetra acetic acid (NM-BAPTA) in the reagent under alkaline conditions to form a complex. This complex reacts in the second step with EDTA (ethylenediaminetetraacetic acid). The absorbance is measured at a 340–376nm wavelength which is proportional to the concentration of calcium in the serum sample. The change in absorbance is directly proportional to the calcium concentration and is measured photometrically at a detection limit of 0.20mmol/L. The expected value for serum calcium level for a normal female adult is 2.15–2.50mmol/L.

Quality control was ensured through commercially available built-in control sera for every batch of sample analysis and the intra- and inter-assay coeffi cient of variation were calculated and compared with manufacturer recommendation ranges according to the laboratory standards. Serum magnesium levels below 0.66mmol/L are categorized as magnesium deficiency while serum calcium levels below 2.15mmol/L are regarded as calcium deficiency [17].

### Statistical analysis

Data from the proforma were transferred into an excel spreadsheet and then into IBM SPSS version 23.0 software for windows (Armonk, NY: IBM Corp) for analysis. The data were summarized, and categorical variables were presented as frequencies and percentages while continuous variables were presented as means (± standard deviation). The associations between the mean serum levels of magnesium or calcium among women with and without uterine fibroids were assessed using the independent sample t-test. Pearson’s Chi-square test or Fishers exact test, where appropriate, was used to determine the association between categorical variables and uterine fibroid status. Spearman’s rank correlations were used to determine the relationship between the uterine size or the number of uterine fibroids and serum levels of magnesium or calcium. Multivariate analysis was performed using the binary logistic regression model with adjustments made for all the possible confounders. Statistical significance was set at *P* < 0.05.

## Results

The mean age of the participants with uterine fibroids (35.6 ± 5.2 years) was statistically different from that of their comparison group without uterine fibroids (28.4 ± 6.3 years), p < 0.001. The recorded incidence peak age range of women with uterine fibroids was 30–39 years. There were also statistically significant differences in the educational status (p = 0.023) and marital status (p = 0.010) of women with and without uterine fibroids, but no differences were recorded in their parity (p = 0.083) and body mass index (p = 0.217). All the study participants had serum magnesium levels within the normal reference range of 0.66–1.07mmol/l), hence we reported no serum magnesium deficiencies but showed a statistically higher prevalence of calcium deficiency in women with uterine fibroids compared to those without uterine fibroids (8.3% versus 2.1%, *p* = 0.049) [Table 1].

**Figure 1:**
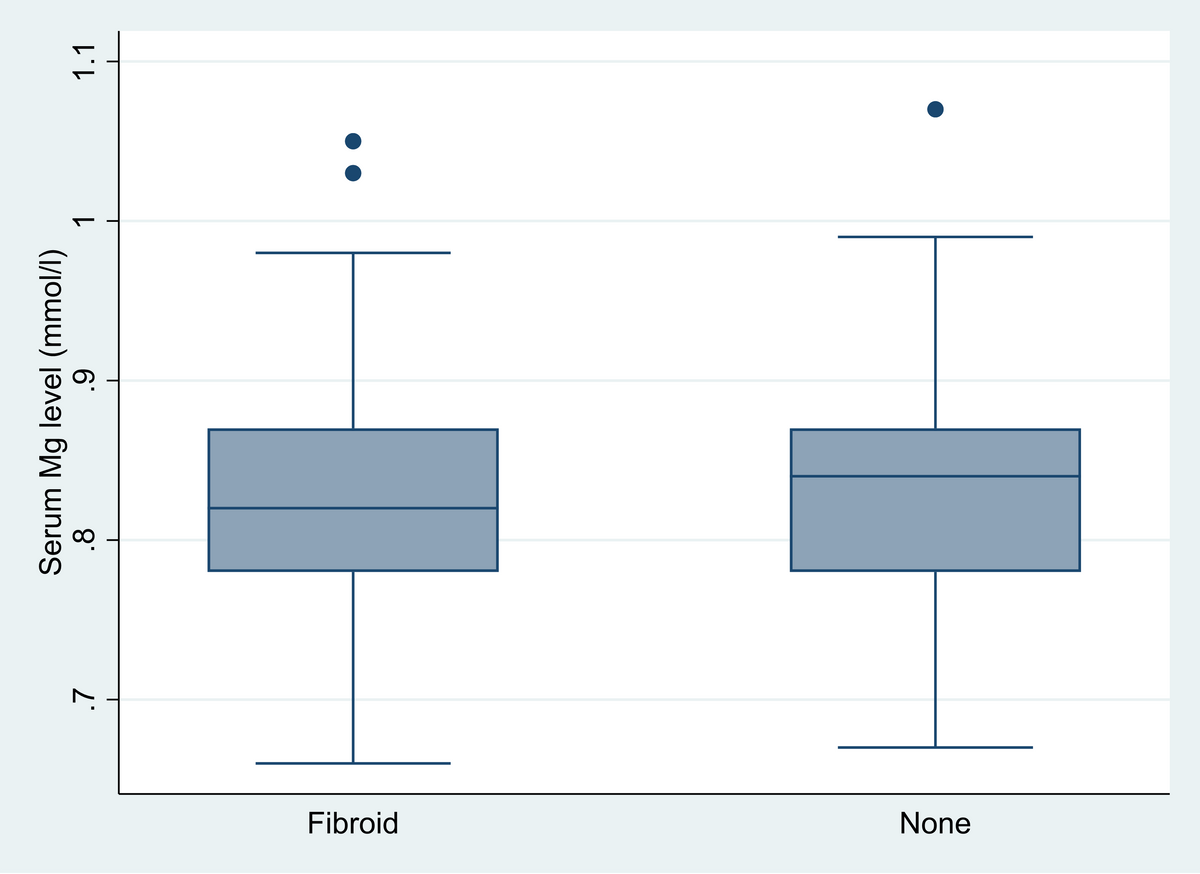
Box plot showing the distribution of serum magnesium levels among women with and without fibroids (0.82 ± 0.07 versus 0.83 ± 0.07 mmol/L, p=0.341).

As shown in Fig. 1, there was no statistically significant difference in the mean serum magnesium levels of women with and without uterine fibroids (0.82 ± 0.07 versus 0.83 ± 0.07 mmol/L, p = 0.341).

**Figure 2:**
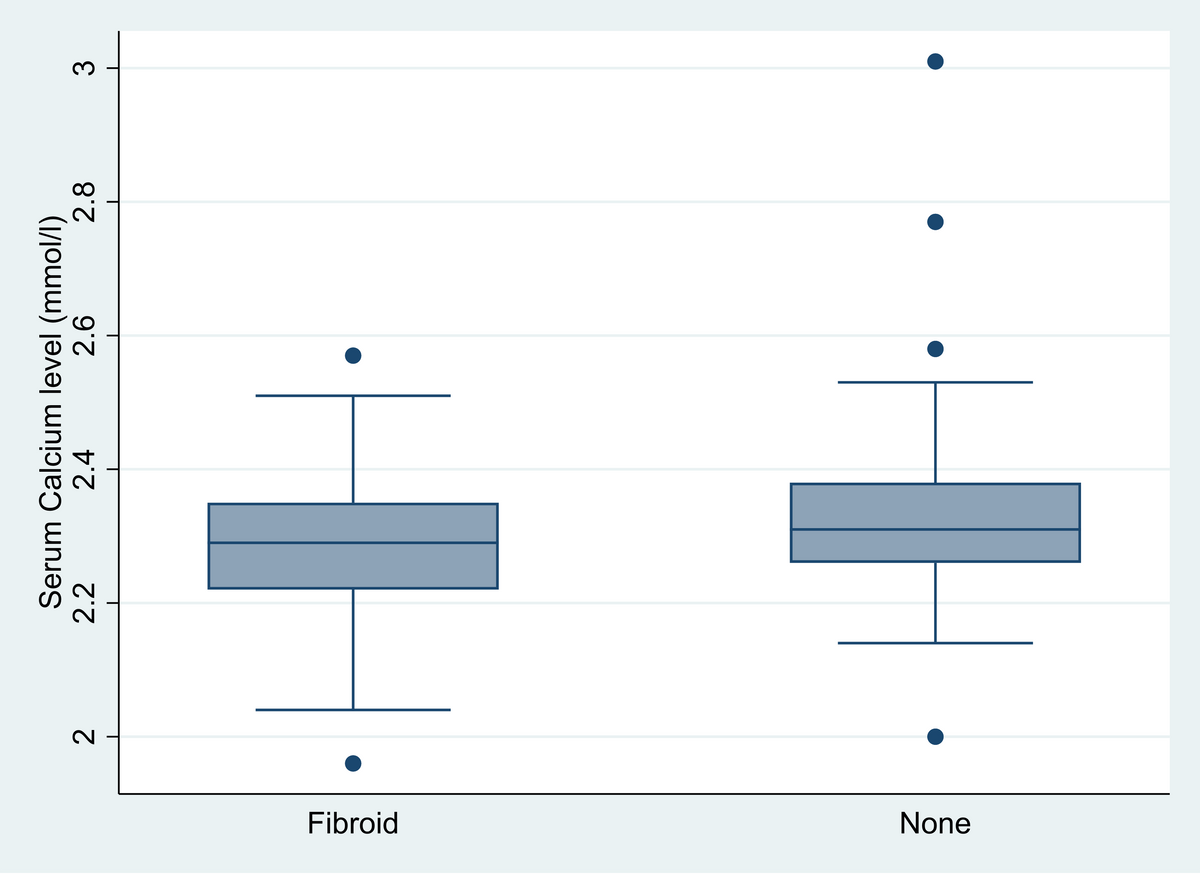
Box plot showing the distribution of serum calcium levels among women with and without uterine fibroids 2.29 ± 0.11 versus 2.33 ± 0.13mmo/L, p=0.012)

However, statistically lower mean serum calcium levels were recorded in women with uterine fibroids compared to their counterparts without uterine fibroids (2.29 ± 0.11 versus 2.33 ± 0.13mmo/L, p = 0.012) [Fig. 2].

Analysis of the serum magnesium-to-calcium ratio revealed no statistically significant difference in women with and without uterine fibroids (p = 1.000). After adjusting for age and educational status in a multivariate analysis, there was a statistically significant association between serum calcium levels and uterine fibroids with about a 94% decrease in the odds of having uterine fibroids for every unit (mmoL) increase in the serum calcium levels (adjusted odds ratio = 0.06; 95% CI: 0.004–0.958; p = 0.047) [Table 2].

Results from binary logistic regression models with participants’ serum calcium levels as the explanatory variable with adjustments made for participants’ age and educational status. Primary/secondary education as reference category

**Figure 3:**
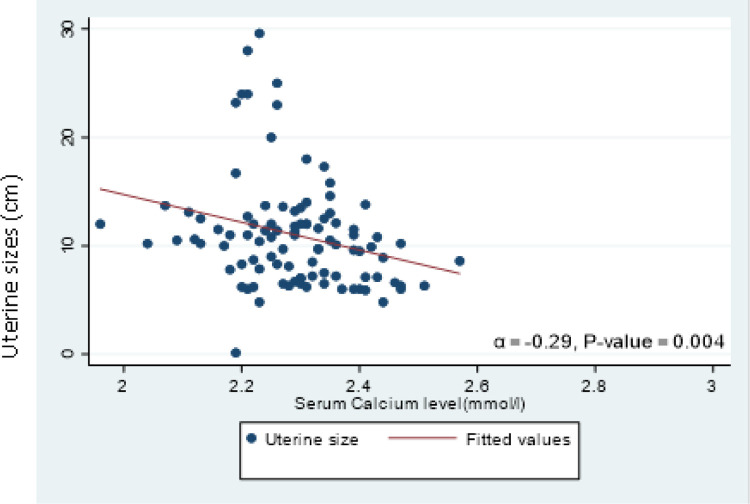
Scatter plot and line of best fit for the correlation between calcium and uterine size (α=−0.29, p = 0.004)

**Figure 4:**
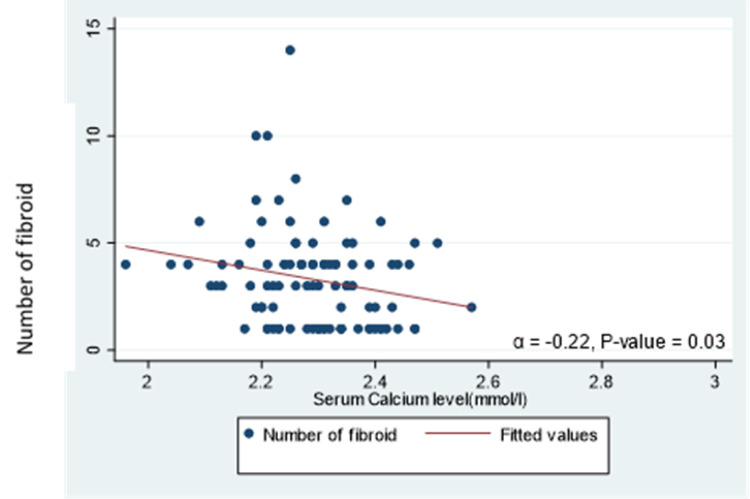
Scatter plot and line of best fit for the correlation between calcium and the number of uterine fibroids (α=−0.22, *p*=0.030)

We recorded statistically significant negative correlations between serum calcium levels and uterine size (α=−0.29, *p* = 0.004) [Fig 3] and the number of uterine fibroids (α=−0.22, *p*=0.030) [Fig 4].

## Discussion

This present study revealed a statistically significant difference in the age, educational status, and marital status of women with and without uterine fibroids. The higher age of women with uterine fibroids and their peak age of 30–39 years corroborated the findings by Isah et al in Abuja [6] and Akinlua et al in Ado-Ekiti [18], Nigeria that reported uterine fibroids as being commoner among reproductive age women in their 3^rd^ and 4^th^ decades of life [6]. This may also be further explained by the high overall age of 32.0 years among participants in this study thus suggesting these urban-dwelling women usually delay getting married and having children earlier due to their rigorous academic and career pursuits [19]. This, therefore, results in prolonged estrogen and progesterone hormone exposure with subsequent stimulation of fibroid growth [6].

Magnesium is responsible for maintaining genomic stability by stabilizing DNA and chromatin, regulating cell proliferation and acting as an enzyme co-factor for DNA processing and removal of the region of DNA damage [20,21], a mechanism that is implicated in the development of uterine fibroids. Conversely, our study revealed no positive association between serum magnesium levels and uterine fibroids, a finding that is consistent with that of the studies by Oyeyemi and co-workers in Ekiti in 2016 [12] and in the same setting by Makwe and colleagues in Lagos in 2021 [7]. We, however, posit that the lack of positive findings in our study and that of Oyeyemi and colleagues [12] does not completely rule out some degree of genomic instability with only a little reduction in the serum magnesium level [22]. The finding of a statistically significant difference in the serum calcium levels in women with and without uterine fibroid in this study is in tandem with the finding by Li et al [11] among Chinese women in 2020 and also in a prospective cohort study by Wise et al in the United States [23] that reported a positive relationship between daily calcium-rich dairy food consumption and uterine fibroids. This relationship is possibly explained by the involvement of calcium in cell cycle regulation in a variety of tumours and oncogenic pathways [24] as the decreased cellular influx of calcium results in reduced myometrial contractility that leads to enhanced proliferation of leiomyoma cells [25,26]. Our finding is, however, in contrast to the findings in the Nigerian studies by Oyeyemi et al [12] and Akinlua et al [18] that reported significantly higher levels of serum calcium in women with uterine fibroids compared to those without. The conduct of a robust longitudinal study and systematic review will add to the body of knowledge in this area by either confirming or refuting the findings of this study.

The altered expression of calcium ion (Ca^2+^) channels and pumps are recognized features of tumorigenesis in certain organs due to their regulation of both cell death and proliferation [27] which is corroborated by the findings of this study where statistically significant negative correlations were recorded between serum calcium levels and uterine size and the number of uterine fibroids. This is equally in addition to the reported finding by Li and co-workers [11] of low serum calcium levels in women with multiple and huge uterine fibroids compared to those with solitary fibroids and smaller fibroids. There are a few limitations in our study and these include the cross-sectional design which made it difficult to infer any causal inferences from the relationships that were reported and the hospital-based setting which limits the ability to generalize our findings to the entire population of women with uterine fibroids.

## Conclusions

This study found significant inverse associations between low serum calcium levels and uterine fibroids, uterine size, and the number of fibroid nodules. However, no significant association was observed between serum magnesium levels and uterine fibroids. These findings suggest the promising role of calcium-rich diets and supplements in the prevention of uterine fibroids among Nigerian women. However, more reliable evidence should be obtained from future longitudinal studies in evaluating the role of these trace mineral levels especially calcium in the development of uterine fibroids.

## Figures and Tables

**Table 1:** Characteristics of the participants with and without uterine fibroids

Characteristics	Fibroids	No Fibroids	Overall	*p-value*
	**n = 97**	**n = 97**	**n = 194**	
**Age (yr) mean ± SD**	35.6 ±5.2	28.4 ±6.3	32.0 ±6.8	0.001
20–29	14 (14.4)	62 (63.9)	76 (39.2)	0.001
30–39	60 (61.9)	29 (29.9)	89 (45.9)	
≥40	23 (23.7)	6 (6.2)	29 (15.0)	
**Educational status**				
Primary education	5 (5.2)	0 (0.0)	5 (2.6)	0.023^^^
Secondary education	22 (22.7)	15 (15.5)	37 (19.1)	
At least tertiary education	70 (72.2)	82 (84.5)	152 (78.4)	
**Marital status**				
Married	64 (66.0)	49 (50.5)	113 (58.3)	0.010^^^
Single	30 (30.9)	48 (49.5)	78 (40.2)	
Divorced	2 (2.1)	0 (0.0)	2 (1.0)	
Widowed	1 (1.0)	0 (0.0)	1 (0.5)	
**Parity**				
Nulliparous	82 (84.5)	72 (74.2)	154 (79.4)	0.076
Multiparous	15 (15.5)	25 (25.8)	40 (20.6)	
**BMI (kg/m** ^**2**^ **) mean ± SD**	25.4 ±5.1	26.4 ±5.3	25.9 ±5.2	0.217
Underweight (< 18.5)	4 (3.0)	2 (3.0)	6 (6.0)	0.487
Normal (18.5–24.9)	50 (48.0)	46 (48.0)	96 (96.0)	
Overweight (25.0–29.9)	26 (25.0)	24 (25.0)	50 (50.0)	
Obesity (≥ 30)	17 (21.0)	25 (21.0)	42 (42.0)	
**Calcium levels (mmol/L)**				
Low (< 2.15)	2(2.1)	8 (8.3)	10 (5.2)	0.049
Normal (2.15–2.50)	88 (90.7)	87 (89.7)	175 (90.2)	
High (>2.50)	7 (7.2)	2(2.1)	9 (4.6)	


Abbreviations: BMI, body mass index (calculated as weight in kilograms divided by the square of height in meters), yr, years.

Values are given as mean ± SD, median (interquartile range), or number (percentage) unless indicated otherwise.

^ Fishers exact test

**Table 2:** Univariate and multivariate analyses of the association between serum calcium levels and uterine fibroids.

	Presence of uterine fibroids			
Factors	Unadjusted		Adjusted	
	OR (95% CI)	*P*-value	OR (95% CI)	P-value
**Serum calcium levels in mmol/L**	0.036 (0.002 – 0.530)	0.016	0.058 (0.004 – 0.958)	0.047
**Age in years**	1.220 (1.1501.290)	0.001	2.007 (0.121 – 2.825)	0.002
**Tertiary**	0.470 (0.230 – 0.960)	0.410	-	-

Abbreviations: CI, confidence interval; OR, odds ratio.

## Data Availability

The datasets generated during and/or analysed during the current study are not publicly available due to privacy of collected participants’ data but are available from the corresponding author on reasonable request.
